# A protocol to analyze the global literature on the clinical benefit of interlimb-coordinated intervention in gait recovery and the associated neurophysiological changes in patients with stroke

**DOI:** 10.3389/fneur.2022.959917

**Published:** 2022-11-17

**Authors:** Shijue Li, Haojie Zhang, Yan Leng, Di Lei, Qiuhua Yu, Kai Li, Minghui Ding, Wai Leung Ambrose Lo

**Affiliations:** ^1^Department of Rehabilitation Medicine, The First Affiliated Hospital, Sun Yat-sen University, Guangzhou, China; ^2^Guangdong Engineering and Technology Research Centre for Rehabilitation Medicine and Translation, The First Affiliated Hospital, Sun Yat-sen University, Guangzhou, China

**Keywords:** interlimb coordinated, stroke, gait, fMRI, limbs coordination, motor recovery

## Abstract

**Background:**

Stroke is among the leading causes of disability of worldwide. Gait dysfunction is common in stroke survivors, and substantial advance is yet to be made in stroke rehabilitation practice to improve the clinical outcome of gait recovery. The role of the upper limb in gait recovery has been emphasized in the literature. Recent studies proposed that four limbs coordinated interventions, coined the term “interlimb-coordinated interventions,” could promote gait function by increasing the neural coupling between the arms and legs. A high-quality review is essential to examine the clinical improvement and neurophysiological changes following interlimb-coordinated interventions in patients with stroke.

**Methods:**

Systematic review and meta-analysis will be conducted according to the Preferred Reporting Items for Systematic Reviews and Meta-Analyses (PRISMA). The literature will be retrieved from the databases of OVID, MEDLINE, PubMed, Web of Science, EMBASE, and PsycINFO. Studies published in English over the past 15 years will be included. All of the clinical studies (e.g., randomized, pseudorandomized and non-randomized controlled trials, uncontrolled trials, and case series) that employed interlimb intervention and assessed gait function of patients with stroke will be included. Clinical functions of gait, balance, lower limb functions, and neurophysiologic changes are the outcome measures of interest. Statistical analyses will be performed using the Comprehensive Meta-Analysis version 3.

**Discussion:**

The findings of this study will provide insight into the clinical benefits and the neurophysiological adaptations of the nervous system induced by interlimb-coordinated intervention in patients with stroke. This would guide clinical decision-making and the future development of targeted neurorehabilitation protocol in stroke rehabilitation to improve gait and motor function in patients with stroke. Increasing neuroplasticity through four-limb intervention might complement therapeutic rehabilitation strategies in this patient group. The findings could also be insightful for other cerebral diseases.

## Highlights

- Human locomotion involves the coordination of all four limbs.
- Interlimb-coordinated intervention is proposed as an effective way to improve gait in patients with stroke.
- The neurophysiological changes and clinical benefits of interlimb-coordinated intervention in patients with chronic stroke remain unclear.
- The findings of this study will offer insight into the neurophysiological adaptations of the nervous system in patients with stroke.

## Introduction

Stroke remains one of the leading causes of adult disability worldwide (1), and the demand for stroke rehabilitation service is likely to continue to grow due to the aging society. Gait dysfunction is common among patients with stroke (2). Approximately two-thirds of the patients have mobility deficits after stroke occurrence (3). A study reported that 1 year after stroke occurrence, half of the stroke survivors could not complete a 6-min walk test, and those who did were only able to perform 40% of the predicted normal distance (4). Gait is essential to safely conduct daily living activities and improve the quality of life, but substantial advances are yet to be made in stroke rehabilitation practice to improve the clinical outcome of gait recovery (5).

The current regime of gait rehabilitation involves high-intensity, repetitive, task-specific intervention (6). Gait rehabilitation may utilize over ground or treadmill with or without body weight support (7). Other interventions such as virtual reality (8), robotics (9), muscle strengthening exercise (10), and electrical stimulations (11) demonstrated various degrees of success in gait recovery. Despite the reported positive outcomes, recent literature proposed that a common gait rehabilitation regime is suboptimal to activate the paretic motor neuron pools due to the training intensity (12). Thus, the clinical outcome is below expectation, raising doubts over the efficacy of traditional gait training in patients with stroke. A new rehabilitation strategy is urgently needed. While increasing the intensity through the increase of step number or training speed may be an appropriate way to improve clinical outcome, it may increase the likelihood of overuse injury or stress fracture due to inefficient walking biomechanics (12). It has also been reported that high-intensity training may only be appropriate for high-functioning people post-stroke (13). In addition, studies that investigated high-intensity exercise training in patients with stroke reported small but non-significant difference in primary motor cortex excitability (14), and no significant difference in corticospinal excitability after high-intensity training (15). These studies casted some uncertainties in the clinical effectiveness of increase training intensity. Neural imaging studies conducted in healthy individuals reported stronger activations in multiple cerebral cortices, including the supplementary motor area (SMA), premotor area (16), and the cerebellum (17), during ipsilateral arm and leg movement in the opposite directions than during ipsilateral arm and leg movement in the same direction and during single-limb movement. It is, therefore, reasonable to expect that patients with stroke who received interlimb-coordinated intervention would have stronger cortical activation than a simple increase in intensity, which theoretically correlates with gait function improvement.

The pendula motion of the upper extremities plays a vital role in gait (18). The role of upper extremities during walking was demonstrated by the alteration of gait pattern after upper limb constraining in healthy individuals and in patients with stroke. These alterations included a reduction in limb coordination (19), spatial parameters of stride length, stride frequency, walking velocity (20), and muscle activation (21). A recent study conducted on healthy participants reported that lower limb muscle activity was driven by the upper limb muscle activity during specific gait phases through the subcortical and cortical pathways to achieve intermuscular coherences of the upper and lower body segments (22). This evidence supports the importance of upper and lower limb coordination in gait recovery. A study on the effectiveness of stationary arm cycling in patients with chronic stroke reported a significant improvement in gait (23), confirming the role of the upper limb in gait function. It was suggested that the cycling motion shared a common locomotion pattern with walking based on the reciprocal lower limb muscle coordination (21). The human gait motion involves specific coordination patterns between upper and lower body segments and requires the synergistic contraction of various muscle groups on the bilateral side (24). Thus, the bipedal human locomotion is built upon the coordination of quadrupedal that involves the coordination of all four limbs (25). The coordination of four limb motion has been coined “interlimb coordination,” where all four limbs move in coordination to accomplish a task, and this has been recently proposed to enhance limb movement control through an increase in the neural coupling between arms and legs (22). The reciprocal lower limb muscle coordination during gait shares some common motor pattern with cycling (26). This theory is given some support by studies that reported significant improvement in gait and lower limb motor function in patients with stroke who underwent arm cycling intervention (23, 27). Other authors proposed that interlimb-coordinated intervention could promote gait function by increasing the neural coupling between the arms and legs (28). This theory is given support by some preliminary data that demonstrated favorable outcomes of interlimb-coordinated training over conventional intervention in patients with chronic stroke (29).

The rhythmic control of gait motion is primarily modulated by the central pattern generator (CPG) and the peripheral sensory feedback that provides the basic synchronous movements of the arms and legs (30). CPG is the functional network of the spinal neurons that regulates the neural coupling of the four limbs at the spinal level during rhythmic task, such as walking and stepping (31). Impairment of the neural coupling of the upper and lower body segments in patients with stroke occurs despite the infarction taking place at the higher cortical level (32). The afferent input processing from the paretic side is impaired which prevents neural coupling, whereas the pathways from the unaffected hemisphere to the unaffected limbs are strengthened after the occurrence of stroke (33). Thus, the motor paresis of contralateral body side to the lesioned brain induces an asymmetry between the right and left limbs, and bimanual coordination required for symmetrical or asymmetrical task performance is impaired (34). Since the lesioned hemisphere may not be able to contribute effectively in voluntarily modifying the motor movement, patients with stroke exhibit incoordination and asymmetry for bilateral to quadri-limb performance which also interferes with the motor and functional recovery (35). The impaired neural coupling also affects the body coordination of patients with stroke, where the non-affected side had to slow down to match the movement of the affected side (36). Other study conducted on patients with chronic stroke also observed the disrupted neural linkage of the affected side in force production during coordinated bimanual task (37). This evidence indicates that despite the primary injury site of stroke being at the cortical level, functional improvement at the spinal-level neural circuit also plays a role in the neural linkage of limbs which contributes to effective gait recovery (23).

The spinal cord itself has central pattern generators and is able to generate coordinated locomotor electromyography (EMG) activity (38). It controls rhythmic movements by producing rhythmic muscle activation without volitional motor control (39). Thus, the locomotor function may be enhanced by accessing the interlimb neural linkage at the spinal circuitry level. The presence of interlimb coupling could be assessed by EMG activity that records the effects of movement in one limb on another limb's muscle activity (40), and also the spinal reflexes that examine the modulation in neural activity associated with the interlimb neural coupling (41). Early literature indicated that arm cycling exercise was able to suppress the hyperexcitability of the soleus which subsequently contributes to improvement in gait (42). A study conducted in patients with chronic stroke reported normalization of cutaneous reflex modulation and increase in soleus stretch reflex amplitudes after arm cycling training. Gait improvement was also observed post-arm cycling training (23). Therefore, enhancing the interlimb neural connectivity of the CPG may be an effective way to improve gait function (23).

To date, there seems to be no systematic review or meta-analysis that investigated the strength of the evidence on interlimb-coordinated interventions, and the neurophysiological and clinical changes induced by such intervention program. A high-quality review examining the clinical improvements and neurophysiological changes following interlimb-coordinated interventions may instigate the establishment of future clinical practice guidelines for clinicians and practitioners. Thus, we present our protocol to critically evaluate the evidence on the change in clinical and neurophysiology measures induced by interlimb-coordinated interventions in patients with stroke. Our review question is “What are the clinical benefits and neurophysiological changes at the cortical and spinal level associated with interlimb coordinated interventions in patients with stroke?”

## Methods and analysis

### Search strategy

The systematic review of the literature will follow the Preferred Reporting Items for Systematic Reviews and Meta-analyses (PRISMA) guidelines. It is registered on the International Platform of Registered Systematic Review and Meta-analysis Protocols (43) (Registration No.: INPLASYL2021100012) and PROSPERO (44) (Registration No.: CRD42021277837). The literature will be searched and retrieved from the following databases: OVID, MEDLINE, PubMed, Web of Science, EMBASE, and PsycINFO. The Boolean operators and search string are as follows: (cerebral vascular accident OR stroke) AND (interlimb coordination OR interlimb coordinated) AND (gait OR walking OR lower limb function) AND (Magnetic resonance image OR MRI OR transcranial magnetic stimulation OR TMS OR neurophys^*^ OR reflex OR electromyography OR EMG). Studies published in English over the past 15 years, from August 2021, will be considered for inclusion.

### Type of participants

Participants with chronic stroke (more than 6 months of stroke onset) (45) who are aged between forty and 80 years old and able to stand with or without assistance will be the focus of this study. Furthermore, participants will not be on medication that affects muscle tone at the time of study enrollment, report of any cardiovascular, musculoskeletal, respiratory, or other chronic diseases.

### Inclusion criteria

The inclusion criteria are as follows:

- Full-text studies published in english 15 years prior to August 2021.
- Studies conducted on individuals aged between forty and eighty with chronic stroke.
- Studies that investigated the neurophysiological changes in patients with stroke, including peripheral nerve stimulation to assess the hoffman-reflex pathway, electromyography to examine the heteronymous and contralateral muscle activity and reflex amplitudes, and neural imaging to assess cortical activities.

### Exclusion criteria

The exclusion criteria are as follows:

- Studies that included unilateral intervention.
- Studies that did not mention the screening of medications that might affect muscle tone. previous study reported spatiotemporal parameters of gait could be improved by releasing the upper extremity spasticity (46). therefore, the present study excluded trials that did not specifically screen the application of spasticity medication in accordance with a published study.
- Studies that did not exclude participants with musculoskeletal (29), cardiovascular, respiratory, or other chronic diseases (47). these exclusion criteria are in accordance with the published literature to minimize potential confounding factors that might influence with clinical outcome of gait.

### Outcome measures

Clinical functions of gait, balance, lower limb functions, and neurophysiologic changes are the outcome measures of interest. These include one of the following measures: spatial–temporal parameters of gait, Berg Balance Scale, Fugl-Meyer motor assessment, H-reflex gain and/or amplitudes elicited *via* nerve stimulation, and EMG signals of muscle activities of the muscle bellies of interest. Neurophysiological data refer to parameters that reflect the properties of neurons, glia, and neural network (48). These include neural imaging data that assess brain network connectivity (e.g., functional magnetic resonance and transcranial magnetic stimulation imaging), brain wave signals that assess the cognitive neural process (e.g., electroencephalogram), and electromyography signals that assess muscle innervation.

### Data management

The retrieved articles will go through a three-stage screening process. The articles' titles and abstracts will be reviewed by two researchers at the first stage to ensure they meet the above inclusion criteria. The pair of researchers then independently screen the full text of each article. Any discrepancy of an article between the two researchers will be resolved by a third reviewer who will act as an adjudicator. For articles where the full text is not available in the databases or on the publisher library, the researchers will attempt to contact the corresponding authors to obtain the full article. The final step will involve the assessment of treatment outcome measures. Studies with eligible outcome measures are to be included in the analysis. A random sample will be extracted for inspection from two senior raters for quality assurance. All of the included articles will then be imported to a reference management system (EndNote 19), and any duplicates will be removed.

### Data extraction

The main data to be extracted and analyzed are the descriptive information of article title, journal title, authors, target population, and host institute. The cohort characteristics of sample size, sex, age, stroke onset duration, and infarction location will be recorded. The methodological characteristics of study design, randomization procedures, intervention type, intervention period, and follow-up will be extracted. The outcome measures regarding clinical functions of gait, balance, lower limb motor functions, and neurophysiological assessment will be recorded and analyzed. A minimum of two independent reviewers will extract and summarize the data from all of the studies.

### Risk of bias (quality) assessment

The quality of all included articles will be assessed by the Mixed Methods Appraisal Tool (MMAT) (49). It is a critical appraisal tool that assesses five different categories of study designs, including qualitative, randomized controlled trial, non-randomized controlled trial, quantitative descriptive, and mixed methods. Five core criteria of each study design are evaluated by the responses of “yes,” “no,” and “can't tell.” This tool is chosen due to its ability to assess the quality of a range of study design. A bias assessment will be conducted by two independent reviewers, and disagreements will be discussed to reach a consensus. A narrative summary of the bias risk will also be provided.

### Strategy for data synthesis

Effect sizes at 95% confidence intervals will be collected to assess the relationships within the data, as well as Cohen's d for estimates of effect size. Quantitative data will be extracted from each article, and a chi-squared analysis will be used to determine homogeneity between observed and expected frequencies. Statistical significance will be set at *p* < 0.05. A narrative synthesis will be written if a meta-analysis is not possible due to the heterogeneity of the studies.

## Discussion

This systematic review will be among the first to provide a comprehensive assessment of the neurophysiological changes and clinical benefits of interlimb-coordinated interventions in patients with chronic stroke. Specifically, we aim to explore the effects of interlimb-coordinated intervention on neural coupling as measured by the H-reflex and EMG muscle activities. Changes in cutaneous reflex and muscle activities could be considered as a proxy of spinal plasticity resulting from interlimb training (47). Reflex excitability of the paretic lower limb muscles is often suppressed due to the decreased influence of the corticospinal tract on reflex excitability (50). Thus, it is possible that interlimb-coordinated intervention may induce adaptive plasticity of the interlimb spinal network. The effects of movement in one limb on another limb's muscle activity reflect the presence of neural coupling at the spinal level (40). It was reported that the neural synchronization may be enhanced by interlimb-coordinated tasks that involve multiple limb movement (29). This evidence suggested that improvement of the neural linkage of limbs at the spinal-level neural circuit may contribute to gait recovery (23).

Another potential underpinning mechanism of interlimb-coordinated intervention is the increase in neural activation of cortices. The activations of the supplementary motor area (SMA), premotor area (16), and cerebellum (17) were found to be stronger during ipsilateral arm and leg movement in the opposite direction than ipsilateral arm and leg movement in the same directions and during single-limb movement. Thus, it is reasonable to expect that interlimb-coordinated intervention may also improve cortical activations which contribute to improvement in gait recovery.

Several studies utilized different functional tasks, neurophysiological tools, and measurements which provided insights on the potential clinical benefits of interlimb coordination (29, 47). However, this may contribute to a high risk of study heterogeneity. A narrative synthesis will be formed if heterogeneity proves difficult for the synthesis of a meta-analysis. The final conclusion regarding the implications of neuroplasticity and clinical outcome following interlimb-coordinated intervention in patients with stroke will be drawn from this systematic review. Limitations will also be discussed in detail. Researchers will be able to use the findings of the review to offer insight into the neurophysiological adaptations of the nervous system in patients with stroke. In turn, clinical decision-making and the future development of targeted neurorehabilitation protocols in stroke rehabilitation to improve motor function would be guided. Increasing neuroplasticity through interlimb-coordinated intervention might complement therapeutic rehabilitation strategies in this patient group, and it could also be insightful for other cerebral diseases. This study meets the criteria for waiver of ethics approval of the hosting institute. We will publish the results of our study in a peer-reviewed scientific journal regardless of the outcome.

## Author contributions

DL, YL, QY, and KL contribute to the data analysis and drafting of the manuscript. MD and WL managed the research and adjudicate any dispute. WL, MD, and QY contribute to funding acquisition. All authors fulfill the four authorship criteria and involved in a specific aspect of the study. All authors had read and approved the final manuscript.

## Funding

This study was supported by the National Natural Science Foundation of China (Grant Nos. 81971224 and 82002375) and the Guangzhou Science and Technology Project (Grant No. 201803010083).

## Conflict of interest

The authors declare that the research was conducted in the absence of any commercial or financial relationships that could be construed as a potential conflict of interest.

## Publisher's note

All claims expressed in this article are solely those of the authors and do not necessarily represent those of their affiliated organizations, or those of the publisher, the editors and the reviewers. Any product that may be evaluated in this article, or claim that may be made by its manufacturer, is not guaranteed or endorsed by the publisher.
